# Comparative Evaluation of Total Antioxidant Capacities of Plant Polyphenols

**DOI:** 10.3390/molecules21020208

**Published:** 2016-02-09

**Authors:** Kristóf Csepregi, Susanne Neugart, Monika Schreiner, Éva Hideg

**Affiliations:** 1 Department of Plant Biology, Institute of Biology, University of Pécs, Ifjúság u. 6, H-7624 Pécs, Hungary; kristofcsepregi@gmail.com; 2 Department Quality, Leibniz Institute of Vegetable and Ornamental Crops, Theodor-Echtermeyer-Weg 1, 14979 Großbeeren, Germany; neugart@igzev.de (S.N.); schreiner@igzev.de (M.S.)

**Keywords:** antioxidants, antioxidant activity assessment, structure-activity relationship, polyphenols, caftaric acid, quercetin-3-glucuronide, quercetin-3-glucoside, grapevine (*Vitis vinifera*) leaves

## Abstract

Thirty-seven samples of naturally occurring phenolic compounds were evaluated using three common *in vitro* assays for total antioxidant activity (TAC) testing: the Trolox Equivalent Antioxidant Capacity (TEAC), the Ferric Reducing Antioxidant Potential (FRAP) and the 2,2-diphenyl-1-picrylhydrazyl (DPPH) radical scavenging assay, in addition to the Folin-Ciocalteu reagent reactivity (FCR). We found that antioxidant hierarchies depended on the choice of assay and applied ANOVA analyses to explore underlying structure-TAC dependencies. In addition to statistically confirming the empirically established connection between flavonoid ring-B catechol and high TEAC or FRAP, new correlations were also found. In flavonoids, (i) hydroxyl groups on ring-B had a positive effect on all four TAC assays; (ii) the presence of a 3-hydroxyl group on ring-C increased TEAC and FRAP, but had no effect on DPPH or FCR; (iii) Phenolic acids lacking a 3-hydroxyl group had significantly lower FRAP or DPPH than compounds having this structure, while TEAC or FCR were not affected. Results demonstrated that any TAC-based ranking of phenolic rich samples would very much depend on the choice of assay, and argue for use of more than one technique. As an illustration, we compared results of the above four assays using either grapevine leaf extracts or synthetic mixtures of compounds prepared according to major polyphenols identified in the leaves.

## 1. Introduction

Plant polyphenols are secondary metabolites with strong antioxidant capacities. Although primarily synthesized for the plants’ own defense against oxidative stress [1,2], these compounds retain the ability to act as antioxidants *ex planta* and thus largely contribute to the pharmaceutical and dietary properties of plant derived food [3]. Consequently, characterization of polyphenols as antioxidants is essential for both plant biology and human nutrition. Flavonoids, as well as many other plant polyphenols, possess a chemical structure ideal for free radical scavenging [4]. Their antioxidant properties include reactivity to a variety of reactive oxygen species [5,6,7,8,9,10,11], as well as metal chelating [12,13]. Due to the large diversity of these compounds, antioxidant properties are generally characterized as total antioxidant capacities (TAC). TAC methods assess antiradical activities using either synthetic free radicals or metal ion, such as Fe^3+^ or Cu^2+^ complexes. There are two mechanisms for the radical scavenging reactions of phenolic antioxidants: a hydrogen atom transfer from the phenolic OH group; and an electron transfer followed by a proton transfer. The most frequently used free radicals are 2,2-diphenyl-1-picrylhydrazyl (DPPH [14]) and the cation radical of 2,2′-azinobis(3-ethylbenzothiazoline)-6-sulfonate (ABTS) of the Trolox Equivalent Antioxidant Capacity (TEAC) assay [15]. The ferric ion reducing antioxidant power (FRAP) assay had been designed to measure the antioxidant potential of plasma [16] and was adapted later to evaluate plant extracts [17]. While TEAC and FRAP are recognized as electron transfer based methods, the chemistry of the DPPH assay is more complex. Evidence was presented for hydrogen atom abstraction [18] and also for electron transfer between polyphenols and the DPPH radical in alcoholic solutions [19]; and most recently a combination of these two reactions, proton-coupled electron transfer was suggested to occur [20].

TAC assays differ from each other not only in substrates, but also in reaction conditions and quantification methods, and the need to standardize antioxidant testing has been identified in food science [21], pharmacognosy [22] and medicinal chemistry [23]. To resolve this problem, Huang *et al.* [23] suggested using the total phenols assay [24,25] which is based on electron transfer from polyphenols to molybdene (VI) of a heteropoly-phosphotungstate-molybdate complex of the Folin-Ciocalteu reagent (FCR [26]). This pragmatic approach was mainly based on the fact that the FCR assay is relatively fast and easy to perform [23]. In another study, Clarke *et al.* measured FRAP, DPPH and FCR of diverse extracts prepared from a variety of tropical plants, and concluded that TAC values were highly redundant, and choose DPPH as the preferred method if one dimensional evaluation was required due a limited availability of samples [22].

The aim of the present study was to investigate how critical is the choice of TAC method when evaluating polyphenol rich samples. We chose four popular assays: TEAC, FRAP, DPPH, and FCR, and compared results over a set of 37 (natural and synthetic) pure test compounds. We believe that this is the first work that shows a comparison of all four techniques and examines possible redundancies. Interchangeability of assays would assume that different techniques assigned higher TAC to compounds belonging to the same polyphenol group. Therefore, we also examined how chemical structures influenced assay results. Structure-activity relationships have already been addressed in a number of publications reporting TAC properties of pure compounds, but these works were either focused on one method, such as TEAC [15,27,28,29] or FRAP [30]; or compared results of two using a specific group of polyphenols only, for example TEAC, FRAP of anthocyanins [31] and TEAC and DPPH of flavonols [32]. The novelty of our approach is the use of statistical analyses to establish correlations instead of observing trends and using the above four methods in parallel over a large data set, including both flavonoids and phenolic acids.

The obtained data set allowed us to address to which extent TAC values of pure test compounds are relevant to antioxidant capacities of plant extracts containing these compounds, for example whether structurally different compounds can lead to an additive TAC in plants. Reports on this issue are contradictory, including both antagonistic and synergistic effects when antioxidants are combined [33,34]. Using extracts made of grapevine leaves with distinct polyphenol profiles and generating equimolar mixtures of test compounds we also verified whether and how minor changes in composition were reflected in results of the four TAC methods.

## 2. Results and Discussion

Phenolic compounds used in this study are listed in Table 1 and Table 2. These were chosen to exemplify phenolic compound groups with documented antioxidant properties. Anthocyanidins were shown to have TAC comparable to those of well-known antioxidants such as ascorbic acid (ASA) or Trolox [28,31]. Flavonoids are the largest and predominant group of plant polyphenols with nutraceutical importance [35,36,37]. Flavonols, the most populous group of flavonoids, are present in most plants and consequently their TEAC [15,27,29,30] and FRAP [30] have been studied extensively. Flavanols found in highly consumed green tea [38] have been analyzed with TEAC and DPPH [32], and principal flavanones in common citrus fruit juices were evaluated using TEAC [39]. Hydoxybenzoic (phenolcarboxylic) acids and hydroxycinnamic acids are two large groups of phenolic acids, distinguished by the chemical nature of the substitute in position-1 of the phenolic ring. Phenolic acids are common in plant leaves [40] and these compounds may also act as antioxidants [41].

**Table 1:** Phenolic acids used in the present study. 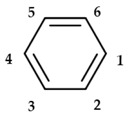

Compound	1	2	3	4	5	6
**Hydroxybenzoic acids**						
2,3-dihydroxybenzoic acid	COOH	OH	OH	H	H	H
4-hydroxybenzoic acid	COOH	H	H	OH	H	H
Gallic acid	COOH	H	OH	OH	OH	H
Syringic acid	COOH	H	OMe	OH	OMe	H
Vanillic acid	COOH	H	OMe	OH	H	H
**Hydroxycinnamic acids**						
Caffeic acid	Acr	H	OH	OH	H	H
Caftaric acid	AcrTa	H	OH	OH	H	H
*p*-coumaric acid	Acr	H	H	OH	H	H
*Trans*-3-hydroxycinnamic acid	Acr	H	OH	H	H	H
*Trans*-ferulic acid	Acr	H	OMe	OH	H	H
*o*-coumaric acid	Acr	H	H	H	H	OH

Abbreviated substitutes: Acr, Acrylic acid; Gal, galactoside; Glc, glucoside, Glu, glucuronide; Me, methyl; Rut, rutinoside; Ta, tartaric acid.

**Table 2:** Flavonoids used in the present study. 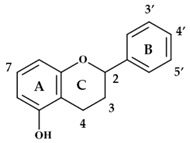

Compound	2–3	3	4	7	3′	4′	5′
**Anthocyanidins**							
Cyanidin	d	H	OH	OH	OH	H	OH
Delphinidin	d	H	OH	OH	OH	OH	OH
Malvidin	d	H	OH	OMe	OH	OMe	OH
Pelargonidin	d	H	OH	H	OH	H	OH
**Dihydroflavonols**							
Dihydrokaempferol	s	2H	=O	OH	H	OH	H
Dihydromyricetin	s	2H	=O	OH	OH	OH	OH
Dihydroquercetin	s	2H	=O	OH	OH	OH	H
**Flavanols**							
Catechin	s	OH	2H	OH	OH	OH	H
Epicatechin	s	OH	2H	OH	OH	OH	H
**Flavanones**							
Hesperetin	s	2H	=O	OH	OH	OMe	H
Hesperidin (Hesperetin-7-*O*-rutinoside)	s	2H	=O	ORut	OH	OMe	H
Naringenin	s	2H	=O	OH	H	OH	H
**Flavone**							
Apigenin	d	H	=O	OH	H	OH	H
**Flavonols**							
Galangin	d	OH	=O	OH	H	H	H
Isorhamnetin	d	OH	=O	OH	OMe	OH	H
Kaempferol	d	OH	=O	OH	H	OH	H
Kaempferol-3-*O*-glucoside	d	OGlc	=O	OH	H	OH	H
Kaempferol-3-*O*-glucuronide	d	OGln	=O	OH	H	OH	H
Kaempferol-3-*O*-rutinoside	d	ORut	=O	OH	H	OH	H
Myricetin	d	OH	=O	OH	OH	OH	OH
Myricetin-3-*O*-glucoside	d	OGlc	=O	OH	OH	OH	OH
Quercetin	d	OH	=O	OH	OH	OH	H
Quercetin-3-*O*-galactoside	d	OGal	=O	OH	OH	OH	H
Quercetin-3-*O*-glucoside	d	OGlc	=O	OH	OH	OH	H
Quercetin-3-*O*-glucuronide	d	OGln	=O	OH	OH	OH	H
Quercetin-3-*O*-rutinoside	d	ORut	=O	OH	OH	OH	H

Ring-C 2-3 bond in flavonoids: s, single bond; d, double bond.

To compare results of various TAC assays, these have to be uniformly calibrated by using the same reference compound. Results of TEAC, FRAP or DPPH assays are usually calibrated using ASA or Trolox, and FCR is traditionally calibrated with gallic acid. The choice of reference compounds has been studied and discussed extensively by Nenadis *et al.* [42] concluding that the choice of standard depended on the aim of the study. For a comparison of relative TAC values, our study required a calibration compound which was (i) a natural product; (ii) a strong antioxidant in all assays; and (iii) gave the same or at least very similar TAC in all assays. With the applied assay conditions (detailed in Materials and Methods), we found that neither ASA nor Trolox met the above conditions.

Among the studied compounds, myricetin-3-*O*-glucoside was the most suitable reference compound for the above requirements, because it gave very similar raw data in the TEAC, FRAP and DPPH assays (within 2.1% relative difference, data not shown). Table 3 gives TAC results using Myr-3-glc as reference. Here, TAC values of Trolox and ASA relative to those of Myr-3-glc are also shown, but these two compounds were not included in the following analyses.

**Table 3:** TAC values of flavonoids and phenolic acids in myricetin-3-*O*-glucoside equivalents.

Compound	TEAC	FRAP	DPPH	FC
**Anthocyanidins**				
Cyanidin	1.267	2.136	1.445	1.245
Delphinidin	2.030	2.043	1.585	1.173
Malvidin	1.402	1.066	0.714	0.942
Pelargonidin	0.998	1.155	0.577	0.597
**Dihydroflavonols**				
Dihydrokaempferol	0.307	0.092	0.029	0.741
Dihydromyricetin	1.256	0.747	1.003	0.603
Dihydroquercetin	0.598	0.962	0.741	0.785
**Flavanols**				
Catechin	1.888	1.255	1.146	1.040
Epicatechin	2.081	1.082	0.178	0.987
**Flavanones**				
Hesperetin	0.659	0.057	0.079	0.869
Hesperidin (Hesperetin-7-*O*-rutinoside)	0.769	0.031	0.016	0.856
Naringenin	0.326	0.000	0.000	0.760
**Flavones**				
Apigenin	0.576	0.000	0.000	0.650
**Flavonols**				
Galangin	0.801	0.161	0.255	0.586
Isorhamnetin	0.718	0.783	0.423	1.145
Kaempferol	0.778	0.852	0.427	0.702
Kaempferol-3-*O*-glucoside	0.598	0.031	0.000	0.491
Kaempferol-3-*O*-glucuronide	0.535	0.021	0.000	0.516
Kaempferol-3-*O*-rutinoside	0.285	0.026	0.000	0.455
Myricetin	2.170	2.105	1.349	1.100
Myricetin-3-*O*-glucoside	1	1	1	1
Quercetin	1.743	2.945	1.470	1.496
Quercetin-3-*O*-galactoside	0.794	1.332	1.130	0.993
Quercetin-3-*O*-glucoside	0.702	1.425	1.067	1.124
Quercetin-3-*O*-glucuronide	0.654	1.281	1.092	1.088
Quercetin-3-*O*-rutinoside	0.688	1.188	1.050	1.132
**Hydroxybenzoic acids**				
2,3-dihydroxybenzoic acid	0.744	1.875	0.784	0.528
4-hydroxybenzoic acid	0.021	0.000	0.000	0.197
Gallic acid	1.903	1.503	1.098	0.535
Siryngic acid	0.875	0.853	0.462	0.278
Vanillic acid	0.511	0.026	0.246	0.391
**Hydroxycinnamic acids**				
Caffeic acid	0.628	1.813	0.763	0.596
Caftaric acid	0.617	1.792	0.852	0.537
*p*-coumaric acid	0.335	0.000	0.011	0.380
*Trans*-3-hydroxycinnamic acid	0.383	0.000	0.000	0.312
*Trans*-ferulic acid	0.759	0.419	0.370	0.508
*o*-coumaric acid	0.574	0.000	0.008	0.397
**Other**				
Ascorbate	0.629	0.666	0.457	0.234
Trolox	0.661	0.903	0.438	0.231

**Figure 1:**
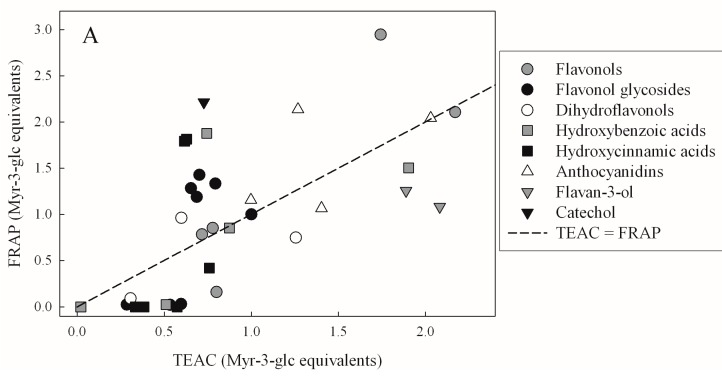

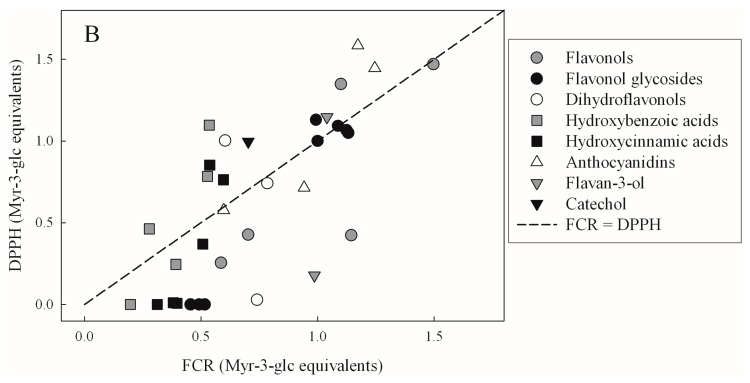
Total antioxidant capacities of various polyphenols as pure test compounds. (**A**) FRAP and TEAC; and (**B**) DPPH and FCR are compared pair wise. Various symbols represent various phenolic compound subgroups according to legends (see Table 1 and Table 2 for a full list of compounds). Dashed lines correspond to the same TAC.

In order to evaluate whether TAC assays favored any compound groups, we made pair wise graphical comparisons (Figure 1). In Figure 1A, TEAC and FRAP values are compared and a dashed line represents equal TAC values. In this way, symbols below or above this line represent compounds larger with TEAC > FRAP or FRAP > TEAC, respectively. A similar graph compares all DPPH and FCR values in Figure 1B, phenolic compounds belonging to the same chemical subgroup are shown using the same symbol and their diversities over the graphs show that the various phenolic compound subgroups did not determine TAC values in our data set.

For example, among flavonol glycosides (represented by filled black circles in Figure 1), some flavonol glycosides showed very low DPPH and FRAP values, while others featured higher ones, more similar to their TEAC. Graphs were similarly inconclusive when TAC assays were paired in different ways (data not shown). The above indicate that differences in TAC values are less influenced by the chemical backbone (main structural elements distinguishing phenolic compound subgroups) than by functional groups attached to the main structure. However, before examining effects of the latter, it should be emphasized that results of the four assays were not independent. Pair wise comparisons of TAC data sets of all 37 studied phenolic compounds were found to be linearly correlated in all possible pairings (Table 4).

**Table 4:** Regression analysis of TAC data sets measured on phenolic compounds listed in Table 1 and Table 2. Regression coefficients *R*^2^ and *p*-values of pair wise linear fits.

	TEAC	FRAP	DPPH
**FRAP**	0.4321	-	-
	*p* < 10^−4^		
**DPPH**	0.4314	0.8137	-
	*p* < 10^−4^	*p* < 10^−4^	
**FCR**	0.3068	0.3951	0.4499
	*p* = 3.76 × 10^−4^	*p* < 10^−4^	*p* < 10^−4^

Table 4 shows that although most method pairings were characterized by relatively low regression coefficients (*R*^2^), all linear regressions were statistically significant at *p* < 0.001. Although its TAC nature is well-recognized [43], FCR is also an established method for assessing the total phenolic content in plant samples. There is an important general consequence of these results which applies to any other experiment with plant-derived samples, and it is to interpret strong correlations between FCR and other TAC (for example TEAC) data sets with care. Statistically significant correlations might simply be due to redundancies in methods, caused by similarities in chemistry (electron transfer from phenolic compounds to chromophores) and do not necessarily indicate that polyphenols are solely responsible for the observed antioxidant properties.

In the following, the two major phenolic subgroups, flavonoids and phenolic acids, are analyzed separately. There is a well-established hierarchy of flavonoids in terms of chemical structure and antioxidant capacities. However, these studies are usually concentrated on one method only. TEAC [4,15,44], DPPH [45,46] and the ability to prevent β-carotene oxidation [46] were shown to be enhanced by the following structural elements in flavonoids: (i) a catechol (3′,4′-dihydroxy) structure in ring-B; (ii) 2,3 double bond in conjugation with a 4-oxo function in ring-C; (iii) the presence of a 3-hydroxyl group in ring-C [47]. Our data set allowed supplementing the above with results of other assays, and statistical analyses to compare how the above factors affected TAC as measured with the four different methods.

The importance of catechol structure to confer high TAC is well-established by studies on the TEAC [13] and DPPH methods [46]. Moreover, deGraft-Johnson *et al.* [48] found that the catechol ring was the only structure positively associated with FRAP. Our data, including 26 flavonoids, of which 13 had at least two hydroxyl groups in ring-B, confirm the above by showing that this catechol structure in ring-B was *p* < 1% significantly coupled to high TAC values of all four methods (Table 5). The novelty of our finding is that compounds with catechol structure had higher FCR as well.

**Table 5:** One way ANOVA testing the null hypothesis that the presence of various flavonoid structures (in rows) as nominal factor had no effect on the specific TAC (in columns).

Structure	TEAC	FRAP	DPPH	FCR
**Flavonoids**				
3′-OH and 4′-OH both present in ring-B *	0.560 ^#^	0.746	0.833	0.654
	*p* = 2.93 × 10^−3^	*p* < 10^−4^	*p* < 10^−4^	*p* < 10^−4^
2,3 double bond and 4-oxo both present in ring-C	*p* = 0.232	*p* = 0.868	*p* = 0.874	*p* = 0.932
3-OH present in ring-C	0.703	0.548	*p* = 0.075	*p* = 0.062
	*p* < 10^−4^	*p* = 3.78 × 10^−3^		
**Phenolic acids**				
3-OH absent	*p* = 0.250	−0.740	−0.670	*p* = 0.057
		*p* = 9.26 × 10^−3^	*p* = 0.024	

* Substituents are numbered according to structures shown in Table 1 and Table 2; ^#^ Values of standardized coefficients are shown when *p* < 0.05 indicated that the null hypothesis was false, and the factor affected TAC.

Ring-C 2,3 double bond in conjugation 4-oxo function was reported to enhance TEAC [49], although Rice-Evans *et al.* [4] suggested that the ring-C 2,3 double bond was less important for high TEAC than the ring-B catechol structure. In another study, DPPH analysis of a large number of flavonoids did not confirm the importance of this double bond in high antioxidant capacities [46]. We tested whether the 2,3 double bond and 4-oxo in ring-C correlated with high TAC values and found no significant connection (Table 5). Our data set included 11 phenolic compounds without and 15 phenolic compounds with both these ring-C characteristics, but the occurrence of only one feature, *i.e.*, the presence of either 2,3 double bond only or a 4-oxo group alone, was less evenly distributed in the data set (18 of 26 or 20 of 26, respectively) making the testing of individual effects unreliable.

Flavonoids are generally present in plants as glycosides, and many as 3-*O*-glycosides. Glycosylated flavonoids were found to have lower TEAC [4] and DPPH [46,50] than their corresponding aglycones. Hydrogen bond between ring-C 3-OH and ring-B hydroxyl groups influences the torsion of the whole molecule, and removal of 3-OH was postulated to lessen electron delocalization capacity via abrogating co-planarity [51,52]. In our data set, 11 of the studied 26 flavonoids had a 3-OH substitution in ring-C, and the presence of this substituent correlated with high TEAC and high FRAP at *p* < 1% (Table 5), confirming the above studies. Effects of ring-C3 substitution on DPPH or FCR were not statistically significant in our data set, although barely failed the threshold (*p* = 0.075 and 0.062, respectively). The striking difference between DPPH assay results of kaempferol and kaempferol-glycosides have already been observed [50], but we found much smaller differences for quercetin and myricetin derivatives (Table 3) which may cause the lack of statistical correlation in the whole data set. Lipophilic free flavonoid aglycones are far less abundant than water-soluble glycosides [53,54,55]. There is no agreement whether the presence of aglycones is due to an arrest of the synthesis or to glycone-aglycone conversions of existing stores, but the presence of aglycones is usually explained by increased need for antioxidant defense, for example during bud development [56]. In our data set, the TEAC assay gave an approximately 50% lower TAC for both kaempferol- and quercetin-3-*O*-glycosides as compared to their aglycones (Table 3). This was also the case when quercetin-3-*O*-glycosides and quercetin were compared using the FRAP or DPPH assays. However, these methods assigned very low, practically zero TAC to kaempferol-3-*O*-glycosides (Table 3). The only exception in our study, when the glycosylation did not result in an increase in flavonoid TAC, was observed in TEAC of hesperetin and hesperidin (hesperetin-7-*O*-rutinoside): the glycosylated flavanone had a 10% higher TEAC than its aglycone form. There was no difference in the FCR of these two compounds—glycosylation lowered both FRAP and DPPH by 45% and 80%, respectively. The difference between the effect of glycosylation on TEAC in flavonols and hesperitin could be explained by differences in ring-C structures, but is more likely due to the fact that hesperidin was glycosylated at its A-ring 7-*O* position and not at the C-ring 3-*O* position as all flavonols in our study. Because hesperetin and hesperidin were the only non flavonol aglycone and glycoside compound pair in our study, we were unable to establish any trend. However, the above result illustrates that the same change in metabolic composition could be interpreted to affect TAC very differently when evaluated using different TAC assays. It also indicates the need of comparative TAC studies using flavonoid-7-*O*-glucosides, although these compounds are difficult to obtain as test compounds.

Another example of various TAC assays evaluating the same change in polyphenol composition differently is the ratio of quercetin- and kaempferol-glycosides. The amounts of these compounds are strongly influenced by growth temperature and light conditions, although to different extents [57], and ultraviolet light reportedly causes a more pronounced increase of quercetin glycosides than of kaempferol glycosides [58,59]. In this way, growth light and temperature provide a tool for manipulating flavonoid compositions in plant-based food. However, different TAC methods would assess the consequences of such changes in metabolites in different ways. Because kaempferol glycosides are practically invisible to DPPH and FRAP (Table 3), these two methods would register increases in quercetin glycosides only, while results of a TEAC assay would account for changes in both quercetin and kaempferol derivatives. This example argues against using the DPPH assay as a stand-alone method to assess TAC [22] in studies either aimed at connections between growth light conditions and flavonoid compositions or performed on medicinal plants rich in kaempferol glycosides [60,61,62].

Although most studies focus on flavonoids as main antioxidants, phenolic acids also contribute to TAC. Structure-antioxidant studies of hydroxycinnamic acids suggest the importance of an unsaturated bond on the side chain and positions of hydroxyl groups on the ring for their TAC [41]. In our data set there were 11 phenolic acids: five hydroxybenzoic and six hydroxycinnamic acids. The side chain structure (whether it was a benzoic or cinnamic acid) had no effect on TAC values, neither phenolic acid group was significantly stronger in any of the four TAC parameters (data not shown). Regarding all 11 phenolic acids as one set, substitutes in almost all other positions were too unevenly distributed for statistical analysis, with the exception of the 3-hydroxyl group which was present in five of the studied phenolic acids compounds and was absent from six. One-way ANOVA showed that the absence of the 3-hydroxyl structure (*i.e.* the presence of either hydrogen or *O*-methyl at this ring position) had a negative effect on FRAP or DPPH, while having no significant effect on TEAC or FCR. This is a new finding, and the fact that FRAP or DPPH assays give equally low (zero or very small) TAC for mono-hydroxy-benzoic acids and coumaric acids (Table 3) may be responsible for the stronger correlation of these two methods over the whole data set than what was observed between other method pairs (Table 4).

**Figure 2:**
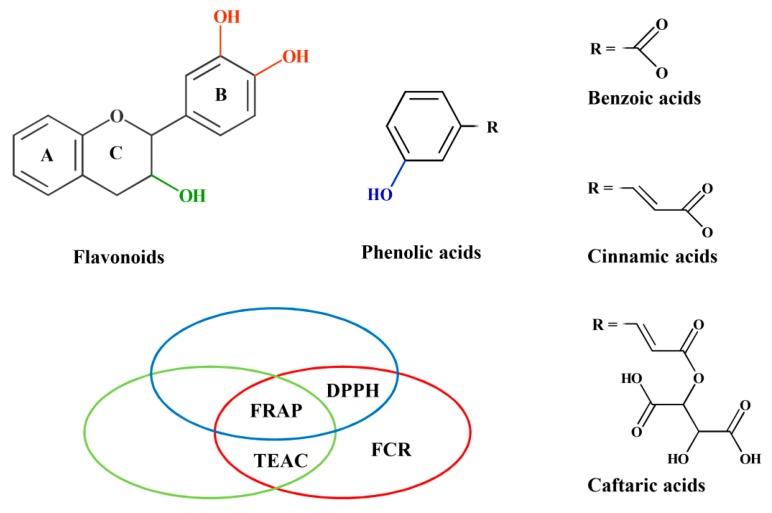
Structure-activity relationships between polyphenol substituents and TAC as found statistically significant in our data set. Hydroxyl groups highlighted in color significantly increased TACs measured with the assays encircled in the same color.

Figure 2 summarizes structure and TAC relationships which were statistically significant in our data sets. Hydroxyl groups highlighted in color significantly increased TACs measured with the assays encircled in the same color. A different influence of the same structural feature on the outcome of different TAC assays argues against relying on a single method when a thorough analysis is required to characterize the antioxidant properties of leaves or polyphenol rich food samples.

To illustrate the above mentioned necessity of multi-angle TAC approaches, we used two sets of grapevine leaf samples. Grapevine leaves are reportedly rich in polyphenols [63,64] and their composition is affected by climate [65]. In our study, *Pinot noir* leaf samples were collected in two consecutive years at the same phase of plant development (see Materials and Methods for details). Phenolic components were determined using HPLC-MS, and we found that leaves in year 2012 contained lower amounts of the same phenolic compounds than in the year 2013 (Table 6). This is probably due to a climate effect: 2012 was a drier year than 2013, total precipitation between budbreak and leaf fall in the vineyard in 2012 and 2013 was 289 and 363 mm, respectively (Péter Teszlák, personal communication).

**Table 6:** Phenolic composition of *Pinot noir* grapevine leaves in two consecutive years.

Compound	Year-2012	Year-2013 (% of Year-2012)
Caftaric acid	1.39	1.60 * (113%)
Kaempferol-3-*O*-glucoside	0.03	0.03 (100%)
Kaempferol-3-*O*-glucuronide	0.04	0.05 (120%)
Quercetin-3-*O*-glucoside	0.98	1.38 * (129%)
Quercetin-3-*O*-glucuronide	3.57	4.23 * (115%)
Quercetin-3-*O*-rutinoside	0.23	0.22 (96%)

Amounts are given as µg per 1 mg leaf dry weight. * Amounts significantly (*p* < 5%) different in 2013 samples from those in 2012 ones in *t*-test.

Correlating climate factors and phenolic compositions was beyond the scope of the present study, but leaves from these two years provided ideal model samples being qualitatively identical, but having small quantitatively differences in composition, which is frequently observed under field conditions. TACs were measured using each of the four assays and all methods identified a significant difference in TAC, but to different extents (Table 7)

**Table 7:** Relative difference in TAC parameters between year-2012 and year-2013 *Pinot noir* grapevine leaf samples.

Compound	TEAC	FRAP	DPPH	FCR
year-2013 average (% of year-2012 average)	120.12%	126.13%	111.50%	132.16%
comparison of year-2012 and year-2013 averages *p* value of paired *t*-test	<10^−4^	<10^−4^	1.55 × 10^−3^	<10^−4^

Although phenolics are abundant in grapevine leaves, these contain an array of other antioxidants, such as ASA, α-tocopherol or β-carotene, which may also contribute to positive TAC assay results. Our first question was whether quantitative changes in phenolics were the main factor behind differences between TAC of the two leaf samples. To test this, two mixtures of pure test compounds (labeled as 2012-mix and 2013-mix) were prepared according to the ratios of main phenolic components identified in leaf extracts as shown in Table 6.

**Figure 3:**
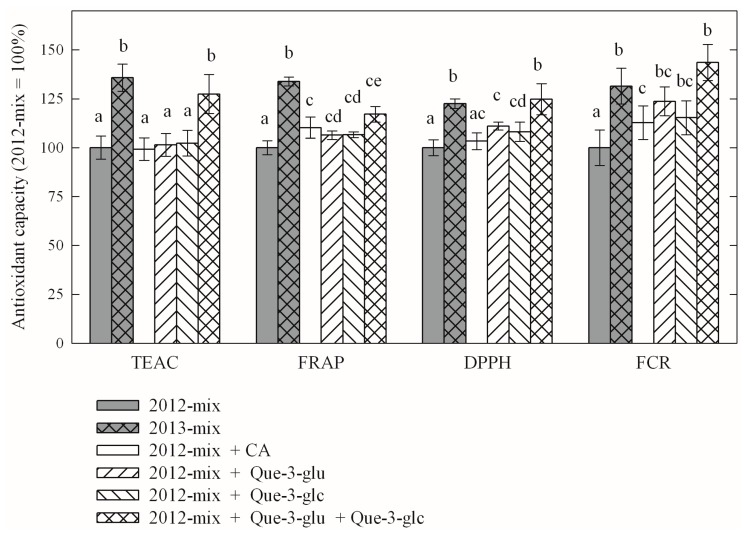
Antioxidant capacities of various polyphenol mixtures. 2012-mix and 2013-mix contain major polyphenols in molar ratios as identified in *Pinot noir* grapevine leaves collected in 2012 and 2013, respectively (see Table 6 for a full list of leaf phenolics). CA, caftaric acid; Que-3-glu, quercetin-3-*O*-glucuronide; Que-3-glc, quercetin-3-*O*-glucoside. Column heights and error bars correspond to means and standard deviations, respectively. Statistically (*p* < 1%) different data sets are labeled using different letters (**a**–**e**).

TAC parameters of these two mixtures were significantly different (compare grey columns with and without crosshatch in Figure 3), corresponding to differences measured in leaf extracts (Table 7). Next, the 2012-mix was complemented with only one of the three major phenolic components caftaric acid (CA), quercetin-3-*O*-glucuronide (Que-3-glu) or quercetin-3-*O*-glucoside (Que-3-glc) to contain an amount characteristic to 2013-mix. One aim of this experiment was to mimic an increase in one of the components only and see whether and to what extent antioxidant capacities were additive, *i.e.*, whether such addition to 2012-mix was sufficient to increase antioxidant capacities to that of 2013-mix. We found that answers to these questions depended on the choice of assay. As shown in Figure 3, the answer to the second question was negative.

An increase in CA alone did not change TEAC or DPPH, but caused a significant increase in FRAP and FCR (empty columns in Figure 3). An effect on leaf FRAP is expected as pure CA has higher FRAP than quercetin glycosides. Increasing the amount of either Que-3-glu or Que-3-glc had no effect on TEAC but increased all other TAC (hashed columns in Figure 3). FRAP and DPPH values of samples complemented in one of the quercetin glycosides were between those of mix-2012 and mix-2013, but this addition was enough to increase the FCR to the level characteristic of 2013 samples (mix-2013). Supplementing the 2012-mix with both quercetin glycosides was sufficient to achieve the TEAC, DPPH and FCR characteristics of the 2013-mix but not the FRAP (crosshatch column in Figure 3).

The above results illustrate the general conclusion of our work, that TAC-based comparisons of phenolic-rich samples may give different conclusions depending on the choice of assay, due to different structure-activity relationships between polyphenol substituents and assay results, as summarized in Figure 2. A practical consequence is to avoid ranking of food or agricultural samples on the basis of a single TAC method only.

## 3. Materials and Methods

### 3.1. Chemicals

Pure test compounds for TAC measurements were purchased from Extrasynthase (Lyon, France) and stored at 4 °C with the exception of apigenin, kaempferol-3-*O*-glucuronide and quercetin-3-*O*-glucuronide which were stored at −20 °C. These compounds were dissolved with aqueous ethanol (70 ethanol:30 water *v*/*v*) and dimethyl sulfoxide. Synthetic leaf mimics were mixed from pure test compounds following molar ratios of major compounds identified in grapevine leaves by HPLC-MS (HPLC:Agilent 1100, Waldbronn, Germany; MS: Bruker, Bremen, Germany).

### 3.2. Plant Material

Leaves from *Vitis vinifera* L. cv. *Pinot noir* were collected from the vineyard of the Institute of Viticulture and Oenology (University of Pécs), from Southern sides of East-West oriented rows. In both years, leaves were harvested at the onset of berry ripening (veraison), five leaves were pooled from each of three different plants and samples were lyophilized. Leaves were collected in two consecutive years, 2012 and 2013 of which the former was drier. For total FCR and TAC measurements, 150 mg of lyophilized leaf samples were extracted into 3 mL hydro-alcoholic solution (H_2_O:EtOH, 30:70 *v*/*v*), centrifuged at 13,000 rpm for 10 min in an Eppendorf minispin centrifuge (Eppendorf Vertrieb, Hamburg, Germany) at room temperature. Supernatants were stored at −20 °C and used for TAC analyses. 

### 3.3. Folin-Ciocalteu Reactivity (FCR) 

Total phenol determination with Folin-Ciocalteu reagent [26] was applied with a modification in incubation temperature as described earlier [66]. 20 µL diluted plant extract or test compound samples were mixed with 90 µL Folin-Ciocalteu reagent (diluted with distilled water 1:10) and 90 µL Na_2_CO_3_ (6% *w*/*v*) in microplate wells. After incubation at room temperature for 90 min, then absorbances were recorded at 765 nm using a Multiskan FC (Thermo Fisher Scientific, Waltham, MA, USA) plate reader. A calibration curve was made using Myricetin-3-*O*-glucoside and FCR values of test compounds were expressed relative to this flavonol. FCR of grapevine leaf samples was given as µM Myr-3-glc equivalent per mg leaf dry weight.

### 3.4. Trolox Equivalent Antioxidant Capacity (TEAC)

2,2′-Azino-bis(3-ethylbenzothiazoline-6-sulfonic cation radical (ABTS^•+^) reduction was measured based on the method of Re *et al.* [15] as described earlier [67]. ABTS^•+^ was prepared by mixing 0.1 mM ABTS, 0.0125 mM horse radish peroxidase and 1 mM H_2_O_2_ in a 50 mM phosphate buffer (pH 6.0). After 15 min, 10 µL diluted leaf extract or test compound was added to 190 µL ABTS^•+^ solution and conversion of the cation radical into colorless ABTS was followed as decrease in absorption at 651 nm. Instead of Trolox, Myr-3-glc was used to prepare a calibration curve and TEAC of test compounds were given in reference to that of Myr-3-glc. TEAC values of grapevine leaf extracts were expressed as µmol Myricetin-3-*O*-glucoside equivalents per mg leaf dry weight.

### 3.5. Ferric Reducing Antioxidant Power (FRAP)

FRAP is based on detecting the capacity of samples to reduce ferric ions, which is measured as an absorbance change of ferrous TPTZ complex. The assay was carried out according to a modification [68] of the original medicinal biochemical assay [30]. The FRAP reagent was prepared by mixing 25 mL of acetate buffer (300 mM, pH 3.6), 2.5 mL TPTZ solution (10 mM TPTZ in 40 mM HCl) and 2.5 mL of FeCl_3_ (20 mM in water solution). For each sample, 10 µL diluted leaf extract or test compound was added to 190 µL freshly mixed FRAP reagent. Samples were incubated in microplate wells at room temperature for 30 min before measuring OD at 620 nm. FRAP values of grapevine leaf extracts were expressed as µmol Myricetin-3-*O*-glucoside equivalents per mg leaf dry weight, FRAP of test compounds were given in reference to that of Myricetin-3-*O*-glucoside.

### 3.6. Determination of DPPH Radical Scavenging Capacity

The assay is based on the loss of violet color of DPPH (2,2-diphenyl-1-picrylhydrazyl) solution when reduced by an antioxidant [14]. For the assay, 75 μM DPPH was dissolved in methanol and mixed with a 100 mM acetate buffer (pH 5.5), 60:40, *v*/*v*. For each sample, 20 µL diluted leaf extract or test compound was mixed with 180 µL of the above solution in a microplate well. After 30 min incubation in dark for 37 °C, the absorption change was measured at 517 nm and DPPH reducing capacities were given in reference to that of Myr-3-glc.

### 3.7. HPLC-MS

Lyophilized leaf samples were ground to fine powder and 0.02 g plant material was extracted three times in a final volume of 1.2 mL of 60% aqueous methanol for 90 min in total. The extract was filtered through Corning^®^ Costar^®^ Spin-X^®^ plastic centrifuge tube filters (Sigma Aldrich Chemical Co., St. Louis, MO, USA), subsequently evaporated to dryness and resuspended in 200 µL of distilled water. Each extraction was carried out in duplicate.

A HPLC series 1100 from Agilent (Waldbronn, Germany) consisting of a degasser, binary pump, autosampler, column oven and photodiode array detector was used to determine the hydroxycinnamic acid derivatives and flavonol glycosides. The extracts were separated on a Supelco Ascentis^®^ Express F5 column (150 mm × 4.6 mm, 5 µm, Sigma-Aldrich Chemie GmbH, Taufkirchen, Germany with a security guard C18 (4 × 4.6 mm, 5 µm, 100 Å) at a temperature of 30 °C using a water/acetonitrile gradient. Solvent A consisted of 99.5% water and 0.5% acetic acid; solvent B was 100% acetonitrile. The following gradient was used for Eluent B 5%–12% (0–3 min), 12%–25% (3–46 min), 25%–90% (46–49.5 min), 90% isocratic (49.5–52 min), 90%–5% (52–52.7 min) and 5% isocratic (52.7–59 min). The flow rate was 0.85 mL·min^−1^, and the detector wavelengths were set at 320, 330, and 370 nm. The hydroxycinnamic acid derivatives and flavonol glycosides were identified as deprotonated molecular ions and characteristic mass fragment ions by HPLC-DAD-ESI-MS*^n^* using an Agilent series 1100 ion trap mass spectrometer in negative ionization mode. Nitrogen was used as the dry gas (10 L·min^−1^, 325 °C) in addition to nebulizer gas (40 psi) with a capillary voltage of −3500 V. Helium was used as the collision gas in the ion trap. The mass optimization for the ion optics of the mass spectrometer was performed for quercetin *m*/*z* 301. The MS*^n^* experiments were performed in auto up to MS^3^ in a scan from *m*/*z* 200–2000. The standards, chlorogenic acid, quercertin-3-*O*-glucoside, kaempferol-3-*O*-glucoside, isorhamnetin-3-*O*-glucoside and cyanidin-3-*O*-glucoside (Roth, Karlsruhe, Germany) were used for external calibration curves (0.01–10 mg per 100 mL).

### 3.8. Data Analysis

Statistical analyses of TAC data were performed using Excel Analysis ToolPack (Version 2007, Microsoft Corporation, Redmond, WA, USA), and XLStat2006 (Addinsoft, New York, NY, USA). Graphs were prepared using SigmaPlot (Systat Software Inc., San Jose, CA, USA).

Pair wise relationships of data sets from different TAC methods were tested using simple linear regression. Linear fits were characterized by the regression coefficient *R*^2^ and *p*-values of *t*-tests. The null hypothesis of these tests was that the slope of the fitted regression line was zero. This hypothesis was rejected for data sets with *p* < 0.05 and these were concluded to be linearly correlated. Actual slope and intercept parameters are not given as these depend on the choice of the assignments of dependent/independent variables (unlike *R*^2^ and *p*) and the purpose of the calculation was to test connectivity between TAC rather than to give mathematical formulae to calculate one TAC from another.

Effects of various structural aspects of the studied compounds on TAC values were evaluated using one-way ANOVA. In this analysis, a chosen structural feature was used as an independent nominal variable (for example, the presence or absence of a certain substitution pattern) and quantitative data from the chosen TAC measurement were used as a dependent variable. The null hypothesis was that the qualitative variable had no effect on the dependent quantitative variable. This was rejected for data sets with *p* < 0.05 and, in this case, the factor was concluded to influence TAC.

TAC values of grapevine leaves were measured using three leaf extracts made from three sets of leaf samples (five leaves each) collected at the same time from different plants. All three leaf extracts were measured five times. In this way, standard deviations from mean values of the two leaf samples (year-2012 and year-2013) represent both biological variability and technical errors of TAC measurements. Synthetic mixtures of compounds were measured in triplicates and in these data sets standard deviations represent technical errors only. Differences between the two leaf samples and differences among synthetic mixtures were tested using paired *t*-tests and TAC values of two data sets with *p*>0.05 were regarded as identical.

## Abbreviations

The following abbreviations are used in this manuscript:

ABTS: 2,2′-azinobis(3-ethylbenzothiazoline)-6-sulfonatic acid, IUPAC name: (2*Z*)-3-ethyl-2-[(*E*)-(3-ethyl-6-sulfonato-1,3-benzothiazol-2-ylidene)hydrazinylidene]-1,3-benzothiazole-6-sulfonate
ASA: ascorbate
CA: caftaric acid, (2*R*,3*R*)-2-[(*E*)-3-(3,4-dihydroxyphenyl)prop-2-enoyl]oxy-3-hydroxybutanedioic acid
DPPH: 2,2-diphenyl-1-picrylhydrazyl
FCR: Folin-Ciocalteu reactivity
FRAP: Ferric Reducing Antioxidant Potential
Myr-3-glc: myricetin-3-*O*-glucoside, 5,7-dihydroxy-3-[(2*S*,5*S*,6*R*)-3,4,5-trihydroxy-6(hydroxymethyl)oxan-2-yl] oxy-2-(3,4,5-trihydroxyphenyl)chromen-4-one
TAC: Total Antioxidant Capacity
TEAC: Trolox Equivalent Antioxidant Capacity
TPTZ: 2,4,6-tripyridin-2-yl-1,3,5-triazine
Que-3-glc: quercetin-3-*O*-glucoside, 2-(3,4-dihydroxyphenyl)-5,7-dihydroxy-3-[2S,3R,4S,5S,6R)-3,4,5-trihydroxy-6-(hydroxymethyl)oxan-2-yl]oxychromen-4-one
Que-3-glu: quercetin-3-*O*-glucuronide, (2*S*,3*S*,4*S*,5R,6*S*)-6-[2-(3,4-dihydroxyphenyl)-5,7-dihydroxy-4-oxochromen-3-yl]oxy-3,4,5-trihydroxyoxane-2-carboxylic acid
